# Solving pain points in guideline development and implementation using human‐centered design

**DOI:** 10.1002/gin2.70014

**Published:** 2025-03-04

**Authors:** Maria Michaels, Chirine Chehab, Mindy Hangsleben

**Affiliations:** ^1^ Centers for Disease Control and Prevention (CDC) Atlanta Georgia USA; ^2^ Chair, Guidelines International Network North America Region (GIN‐NA) Santa Monica California USA; ^3^ Former HHS Entrepreneur‐in‐Residence (Lean Innovation Fellow) US Department of Health and Human Services – Office of the National Coordinator for Health IT (ONC) Washington Washington DC USA; ^4^ Former HHS Entrepreneur‐in‐Residence (Lean Innovation Fellow) US Department of Health and Human Services – Centers for Medicare & Medicaid Services (CMS): Quality Measurement and Health Assessment Group Baltimore Maryland USA; ^5^ Varyn Consulting LLC Saint Louis Park Minnesota USA

**Keywords:** computable, guidelines, guidelines international network, human‐centered design, user‐centered design

## Abstract

Guidelines International Network North America expanded on ‘Adapting Clinical Guidelines for the Digital Age’, an initiative to innovate guideline development and implementation leveraging technology. The objective: solve pain points – problems occurring at different levels of the customer experience – in guideline development and implementation using human‐centered design (HCD). In HCD, customers are people who would use the product or system being designed and are at the center of the design process. HCD builds empathy to understand challenges facing different customers. HCD has three phases: inspiration, ideation, and implementation. *Inspiration phase*: Semi‐structured interviews collected multiple perspectives within guideline development and implementation and determined key pain points (38 participants). *Ideation phase*: HCD workshop ‘Bringing Guidelines to the Digital Age’ defined design goals, determined design criteria, and developed ‘prototype’ solutions for each pain point with feasibility testing during the workshop (35 participants). *Implementation phase*: Postworkshop participants continued developing and testing solution designs, tracked progress, and created products to be further tested, iterated, or published (44 participants). Interviews revealed five pain points in implementation stemming from issues in guideline development: (1) lack of clarity of ‘how’ to apply recommendations; (2) inadequate or nonexistent feedback and testing; (3) unclear language; (4) incomplete information; and (5) lack of informatics expertise. Each workshop group produced prototypes – early models of products built to test a concept or process – that were consolidated into five solution categories and refined through post‐workshop teams focusing on: (1) patient preferences in recommendations (2) real‐world testing and feedback, (3) standard operating procedures for computability and better implementability, (4) artificial intelligence approaches, and (5) connecting informatics with guideline development. HCD provided a user‐centered, iterative approach to design solutions leveraging technology to solve paint points and improve implementation of guidelines into patient care. This is a blueprint for using HCD in guideline development.

## Highlights

- This is a methods paper describing how (1) Guidelines International Network North America (GIN‐NA) applied human‐centered design (HCD) in guideline development and implementation and (2) GIN regional communities, workgroups, or similar groups can model GIN‐NA's approach to connect, learn about, and develop multi‐perspective solutions.
- A multidisciplinary and multi‐organizational group of almost 90 GIN‐NA members applied HCD to develop solutions to address prioritized pain points in guideline development and implementation.
- Throughout this HCD effort, participants expressed improved understanding of other perspectives, thinking differently about their daily work, and planning to apply HCD to improve products they develop.
- Challenges included participants' competing priorities and vacations and relatively short timeframe for the overall effort.

Since 2018, U.S. Centers for Disease Control and Prevention (CDC) led ‘Adapting Clinical Guidelines for the Digital Age’ (ACG) to develop and implement guidelines more easily, quickly, accurately, and consistently.^1^ ACG used a Lean methodology called Kaizen – mapping current state, showing unnecessary redundancy and silos throughout the process, and identifying wastes that participants had control to change and remove in the future state.^2^

The proposed future state redesigned guideline development and implementation, incorporating perspectives of guideline developers, informaticists, implementers, communicators, and evaluators from the beginning. ACG workgroups produced standards, processes, and tools to iteratively codevelop, implement, and evaluate narrative and computable guidelines to decrease time from evidence to practice (i.e., Health Level Seven Fast Healthcare Interoperability Resources [HL7® FHIR®] Clinical Guidelines—also known as CPG‐on‐FHIR®—health information technology standard, integrated process for co‐developing and implementing guidelines, and evaluation framework for the integrated process).^1^, ^2^, ^3^, ^4^, ^5^, ^6^ World Health Organization's (WHO's) SMART Guidelines leverage ACG products (e.g., CPG‐on‐FHIR®) to incorporate computable guidelines to improve implementability across countries.^7^ These standards‐based approaches allow electronic health record (EHR) developers or healthcare organizations to reuse rather than recreate tools that facilitate guideline implementation.

Guidelines International Network North America (GIN‐NA), a GIN regional community, expanded on ACG, identifying and solving pain points in guideline development and implementation using human‐centered design (HCD). Pain points are problems occurring at different levels of customer experience: interaction, customer journey, or relationship.^8^ In HCD, customers are people who use a product or system (e.g., guidelines) and are at the center of the design process. This is a methods paper describing the GIN‐NA HCD effort as a blueprint.

## 1 HUMAN‐CENTERED DESIGN (HCD)

**Figure 1:**
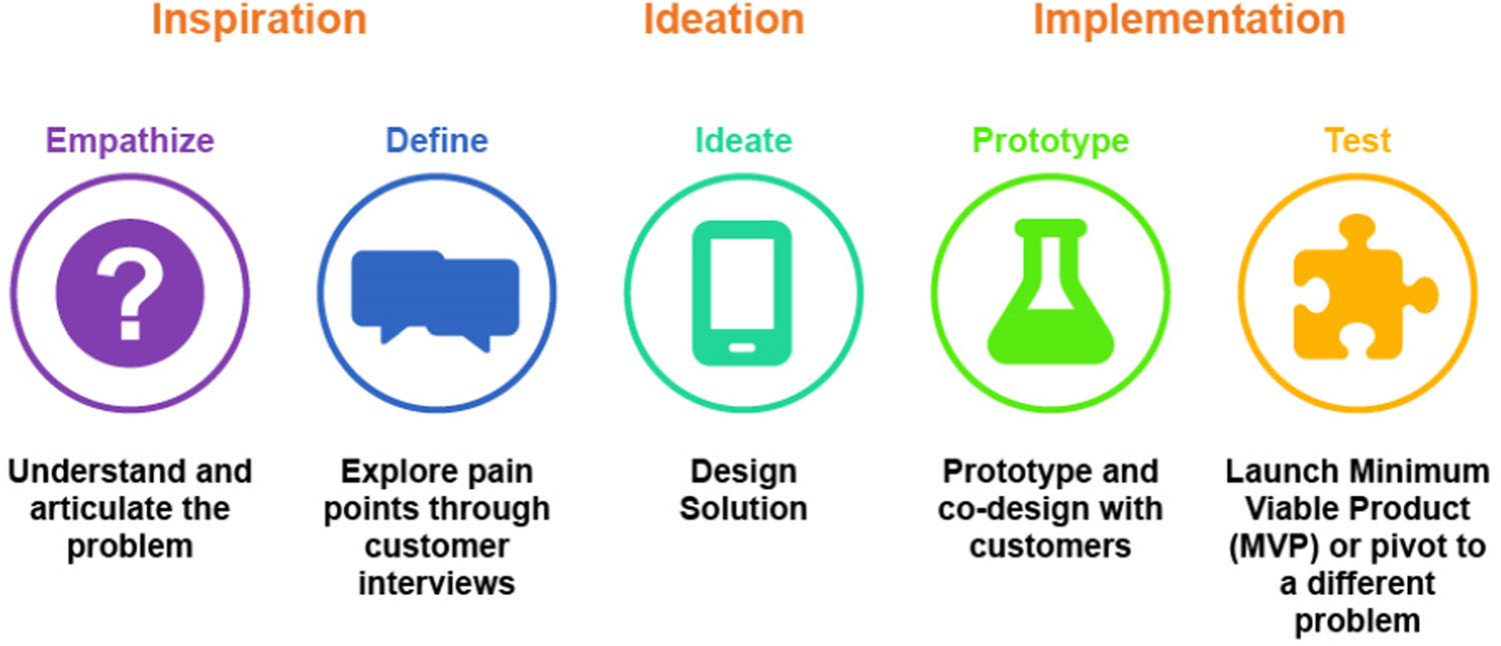
Human‐Centered Design (HCD) Process (adapted from HCD design process by IDEO^11^).

HCD starts with describing customers and their stories and builds towards solutions.^9^, ^10^ HCD is a creative approach to problem solving with three phases: Inspiration, Ideation, and Implementation (Figure 1).^9^, ^11^ ‘Inspiration’ builds empathy with customers by observing their journey and understanding desires and needs. ‘Ideation’ synthesizes findings, identifies pain points, and develops possible solutions through codesign. ‘Implementation’ tests and refines possible solutions into more desirable, feasible, and viable solutions that suit customers' needs or identifies the need to pivot if the wrong problem was being solved. If a different problem is identified, the HCD process iterates with a new three‐phase approach.

In HCD, a ‘product’ is the result of the user‐centered design activity, and ‘prototype’ is an interim product that is iteratively tested to get to a usable product.^12^ HCD has been increasingly used in health research and innovations at the organizational level but has not been broadly applied in guideline development until this effort.^10^, ^13^, ^14^ Each group in this GIN‐NA HCD effort produced different products, some of which are prototypes or designs that were pitched to other organizations to take on further development and others resulted in guidance or tools published separately.

## 2 HCD WORKSHOP ON ‘BRINGING GUIDELINES TO THE DIGITAL AGE’

GIN‐NA Steering Group, composed of 10 GIN members from North America with diverse guideline‐related expertise, launched a 1‐day in‐person workshop in April 2023. GIN‐NA Chair and an HCD expert, both involved in CDC's ACG initiative, led and designed the workshop. The purpose was to operationalize outputs of ACG through solutions that address pain points from multiple perspectives in guideline development and implementation.

A total of 87 unique guideline development and implementation customers participated across three HCD phases, including 23 who participated in two of three phases and 8 who participated in all three phases (Table 1).

**Table 1:** Summary of guideline development and implementation customers by HCD phase.

	Inspiration	Ideation	Implementation
Number of engaged customers	38	35	44
Recruitment source	GIN‐NA Steering Group Members	GIN‐NA Members	GIN‐NA Members
Customer types represented	Patients, Clinicians, Guideline Implementers, Guideline Developers, Informaticists, EHR Vendors, Supporters, Insurer		

### 2.1 Inspiration phase: Pre‐workshop

The GIN‐NA Steering Group created a design brief describing the overarching problem, users and stakeholders, project scope, constraints, exploration questions, expected outcomes, success metrics, and research plan. To capture diverse perspectives, semi‐structured interviews and observations explored insights about guideline development and implementation via video communication with 38 customers: patients (4), clinicians (5), guideline implementers (9), guideline developers (10), informaticists (4), EHR vendors (2), supporters (primarily those managing knowledge repositories) (3), and an insurer.

The interviews' purpose was to inform the development of the workshop and foster empathy with each customer, which met the exemption criteria in The Common Rule.^15^ Interview guides included a verbal consent to proceed, questions on customer experiences, involvement, goals, and barriers to those goals, discussed in 30–60 min, and recorded for notetaking. No identifiers were documented, and recordings were deleted after confirming completeness of notes.

**Figure 2:**
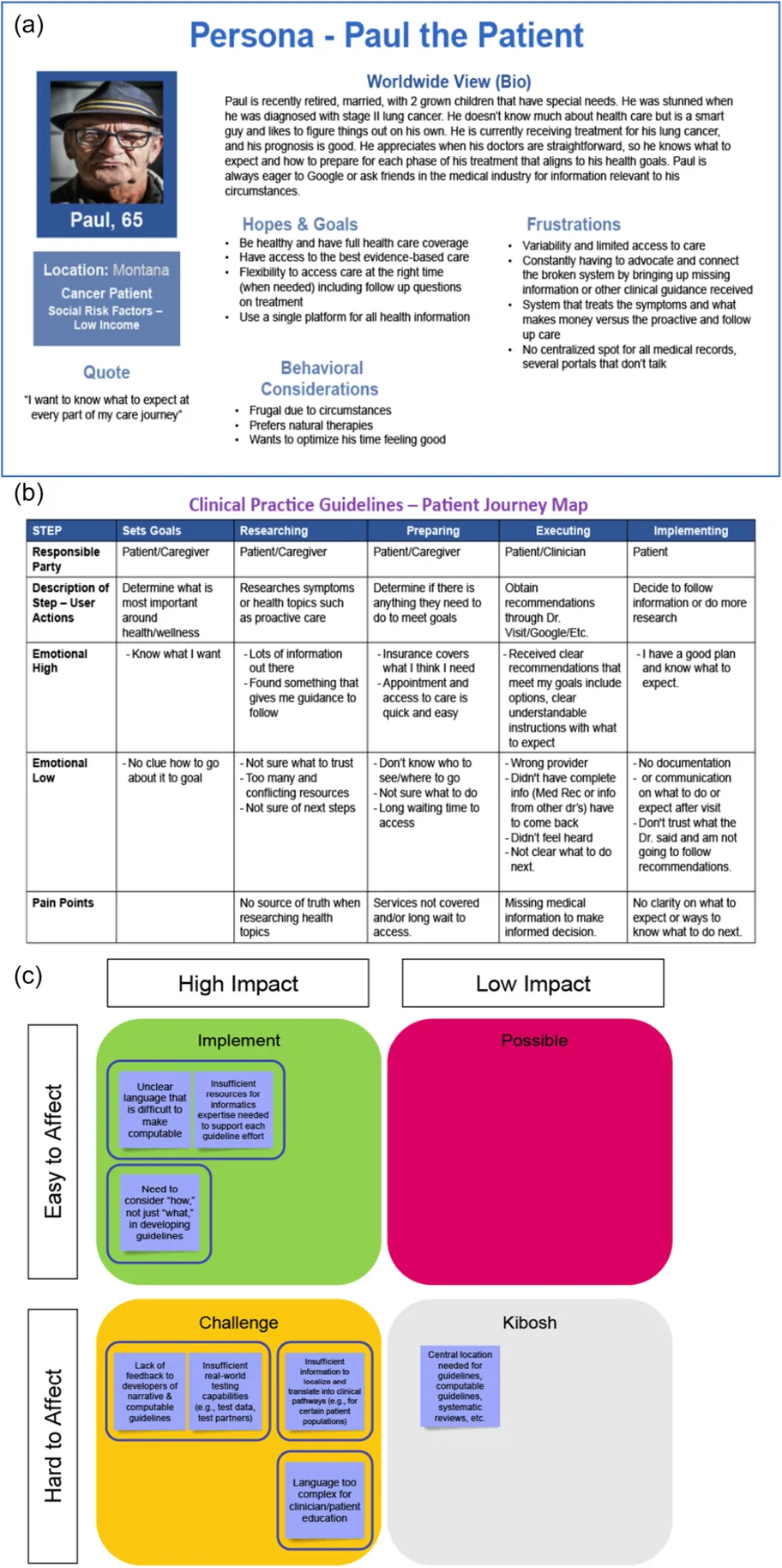
Examples illustrating aspects of the Inspiration Phase: (a) Persona, (b) Journey Map, (c) Possible, Implement, Challenge, and Kibosh (PICK) Chart.

Interview notes were inductively analyzed by the authors, capturing needs, behaviors, and goals towards the problem from different perspectives. Five personas of distinct customer types – patient, clinician, guideline developer, implementer, and informaticist – were created. Using information documented during the interviews, journeys of each customer type were mapped to provide visual representation and facilitate understanding of their experience, emphasizing pain points in task completion. Example patient persona and journey map are in Figures 2a and 2b, respectively. These artifacts were used before the workshop to identify common pain points and during the workshop to illustrate how pain points affect each customer type, informing solution ideation.

GIN‐NA Steering Group used the Possible, Implement, Challenge, and Kibosh (PICK) Chart as a decision‐making tool, prioritizing pain points based on how hard or easy to affect change and impact if implemented (Figure 2c).^16^ GIN‐NA Steering Group selected ‘implement’ (easy to affect, high impact) and ‘challenge’ (hard to affect, high impact) pain points. No pain points were categorized as ‘possible’ (easy to affect, low impact). The pain point categorized into ‘kibosh’ was not selected. Pain points within selected quadrants were consolidated (Figure 2c) and summarized below:

1. Lack of clarity of ‘how’ to apply recommendations.
2. Inadequate or nonexistent feedback and testing.
3. Unclear language for front‐line clinicians or patients.
4. Incomplete information (e.g., for certain patient populations).
5. Lack of informatics expertise to clarify language.

### 2.2 Ideation phase: During‐workshop

The workshop provided background on CDC's ACG initiative, introduced HCD, defined design goals, determined design criteria, and developed prototype solutions with initial testing (co‐designing) among groups. During the workshop, 35 participants representing personas identified in the inspiration phase were divided into groups to design solutions addressing each pain point: (1) ‘How’ + ‘What’, (2) Testing and Feedback, (3) Unclear Language, (4) Incomplete Information, and (5) Informatics Expertise. Groups familiarized themselves with insights from interviews pertaining to their assigned pain point, building empathy informing their designs, then created design goals as ‘*How Might We*…’ statements framing the problem and design criteria based on customers' needs.

Groups brainstormed possible solutions, considering ideas easiest to use, simplest to implement, and most out‐of‐the‐box. Each group created low‐fidelity (i.e., inexpensive, quick representation of an idea or solution concept that has minimum visual details and functionality but enough content to effectively communicate the concept to others) prototypes of their top three ideas. During codesign sessions, individuals visited other groups to learn about their ideas, ask questions, and give feedback on what they liked or wished would be different. Groups incorporated feedback and created plans to further test ideas and determine feasibility to advance to the next iteration. Table 2 summarizes ‘*How Might We*…’ statements, design criteria, and example prototype for each group.

**Table 2:** Design challenges and example prototypes from GIN‐NA HCD workshop groups.

	Design challenge	Prototypes
‘How’ + ‘What’	*How might we* help physicians better understand and adopt the recommendations to improve patient outcomes?	Add informaticist/implementation scientist to guideline panel 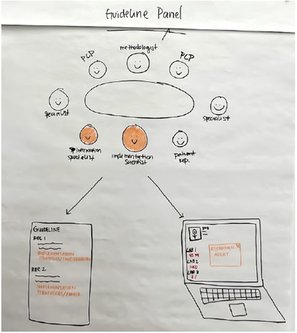
	*Design Criteria:* Use evidence to guide the specific method of implementation Include patients and physicians on a decision team Educate physicians in guideline methods so they feel better equipped to implement guidelines appropriately	
Testing and feedback	*How might we* help guideline developing organizations elicit feedback on patient level data that can be used to update guidelines so that it will improve their quality and relevance?	Create real‐world data feedback loop 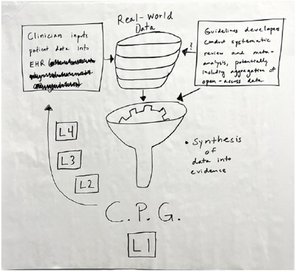
	*Design Criteria:* Collaborative and inclusive Standard‐based artifacts Reusable and shareable across systems, for example, definitions and concepts Minimizes impact of biases User‐centered Collects real‐world data Rapid update mechanism	
Unclear language	*How might we* develop clearer language for implementation by end users (informaticists, patients, clinicians) so that they are computable?	Develop SOP/templates, such as a structured template for developing and wording recommendations. 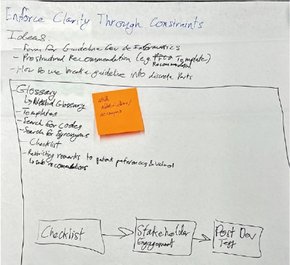
	*Design Criteria:* Clearly define components of narrative recommendations (L1) Discrete Parts Testing before implementation to discern proper interpretation Informaticist in the chain of development (Evidence to Decision) or review before publication (someone to challenge the grey and make it more black and white) Use evidence to guide the specific method of implementation Include patients and physicians on a decision team	
Incomplete information	*How might we* help patients use customized digital tools to make confident evidence‐based decisions that aligns with their values, goals, circumstances, and individual characteristics?	Develop an open‐source method using generative AI to obtain shareable patient generated data 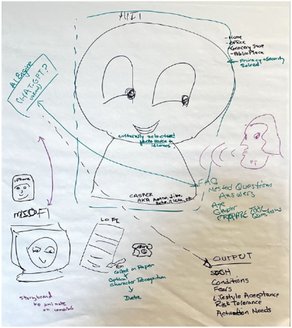
	*Design Criteria:* Accessible Co‐created (opportunities for public comment, crowd source one tool, real world piloting) Communication of applicability Interactive/bidirectional Alert users of new evidence Understandable presentation of options Patients opt into data sharing from other apps Targeted patient questions SDOH, values, goals, preferences, and psychosocial factors Empowers patients to know what to expect, and have easy access wherever they may be Meet Ethical artificial intelligence (AI) principles/standards/considerations Address bias in algorithms to ensure health equity Quality and structure of data that feeds/teaches AI (data integrity)	
Informatics expertise	*How might we* help improve interactions/engagement of guideline developing organizations, guideline developers, and informaticists to advance the understanding of the need for, value and need of producing computable/digitized guidelines?	Create a marketplace that crowdsources resources (e.g., training materials, standards) to create computable guidelines and connects informatics expertise with guideline developers. 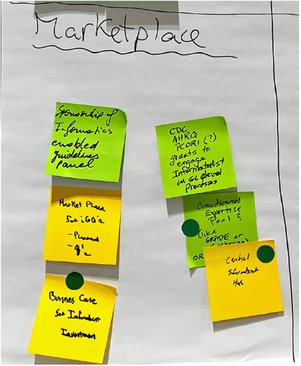
	*Design Criteria:* Follow standards and best practices that provide feedback to make improvements Resources that engage informatics and guideline developers to understand when and how to utilize computable guidelines when needed (what, why, and how) Centralized location that is applicable for all involved in computable guidelines Clear understanding of what competencies informaticists have and when you would use each competency based on need Meets organizational goals and priorities	

### 2.3 Implementation phase: Post‐workshop

Following the workshop, 44 participants (25 workshop participants plus 19 who joined post‐workshop) refined solution ideas developed during the workshop. Similar ideas were consolidated, creating five new teams focusing on each solution type. Team leads volunteered and each participant self‐identified the team(s) to contribute to. For those unable to join the workshop, the initial 3 weeks (1 h per week virtual meetings) involved level‐setting about HCD and how ideas evolved into postworkshop teams. Therefore, teams needed additional time to finish developing and testing their solutions, extending the planned 10 weeks to 16 weeks (May to August 2023).

Teams collaborated to gain insights, research, codesign with other teams, and refine initial solutions to share with external groups to use or advance further, following the iterative nature of HCD. GIN‐NA Chair and HCD expert that led the workshop also facilitated the post‐workshop effort, providing guidance to teams in using HCD for solution refinement and assistance connecting with external groups or individuals with needed insights, occasionally resulting in those individuals joining as participants. They provided tools and templates and helped with logistics, like meeting setup and online collaborative spaces (e.g., Google folders, Miro Boards).

Along with weekly team meetings, biweekly meetings among all teams shared progress, identified help needed, received feedback, and learned more about HCD. Collaborative spaces accessible to all participants and open meetings fostered transparency across groups. Teams pitched solutions to groups that may further develop or pilot the solutions or drafted manuscripts for journal submission.

#### 2.3.1 Patient‐facing/patient‐generated recommendations team

The Patient‐facing/Patient‐generated Recommendations Team focused on ‘*How might we* help patients use customized digital tools to make personalized evidence‐based decisions that align with their values, goals, circumstances, and individual characteristics to increase trustworthiness and foster improved patient outcomes?’ They reviewed the United States Core Data for Interoperability (USCDI) data classes and elements to determine how or which may facilitate patient‐centric computable recommendations.^17^ They identified user journeys: (1) guideline developers create standardized reusable content leveraging reusable data classes and elements, (2) informaticists translate content into standards‐based computable artifacts, and (3) system implementers integrate content into clinician‐ and patient‐facing tools, customizing based on the patient's situation, needs, values, and preferences.

These user journeys translated into two pilot proposals: (1) incorporate reusable data classes and elements (such as those in USCDI) into new and updated guidelines to enable patient‐facing or patient‐generated recommendations, and (2) use artificial intelligence (AI) to leverage real‐world data to identify where inequity exists and ‘reverse engineer’ recommendations that include reusable data classes and elements. The hypothesis for both proposals is that this approach will provide more robust information for shared decision‐making to improve equity in patient care, outcomes, and experience. The team pitched their proposals to the Agency for Healthcare Research and Quality (AHRQ) to execute within programs under their purview. AHRQ is considering the information provided and may request additional discussion as needed.

#### 2.3.2 Real‐world testing and feedback team

The Real‐world Testing and Feedback Team focused on ‘*How might we* help guideline development organizations elicit feedback on patient‐level data that can be used to update guidelines to improve their quality and relevance.’ They refined solutions, working with experts from the HL7® FHIR® community, developing three pilot proposals:

1. Collect user feedback on guidelines being developed or updated.
2. Build queries and engage the community in data exploration activities (before development).
3. Build queries that execute against appropriate real‐world data sources to refine expression of guidelines (during development).

The first proposal is modeled after a feedback mechanism using JIRA for electronic clinical quality measures from the U.S. Centers for Medicare and Medicaid Services that resulted in identifying measure defects earlier, improving quality of measure specifications, and reducing time to resolution across quality reporting programs. The hypothesis is that utilizing feedback mechanisms provides real‐time feedback to guideline developers to iterate during development or maintenance of guidelines or make more robust, usable computable guidelines. The team pitched this proposal to WHO SMART Guidelines team and Infectious Diseases Society of America (IDSA).

The second and third proposals focused on testing during guideline development around patient population, intervention, comparison, and outcomes (PICO) question generation. The second proposal builds queries and engages the community in activities such as connectathons (events testing interoperability of systems) to optimize expression of narrative and computable guidelines.^18^ The third proposal builds queries that execute against appropriate real‐world data sources to refine guideline expression. The hypothesis is that running these real‐world data queries speeds the development and increases guideline quality and uptake. The second and third proposals were pitched to WHO SMART Guidelines team, members of CDC's Division of Global HIV and TB (DGHT), IDSA, National Committee for Quality Assurance (NCQA), and multiple branches from CDC's Division of Cancer Prevention and Control (DCPC). The team prepared use cases to test the solution in HL7® connectathons.^19^

#### 2.3.3 Standard operating procedures (SOP) team

The SOP Team focused on ‘*How might we* help guideline developers write clearer language for implementation by end users (clinicians, patients, informaticists) so that guidelines are consistently translated and implemented.’ They reviewed the GIN‐McMaster Checklist, Institute of Medicine ‘Guidelines We Can Trust’, and CPG‐on‐FHIR® knowledge level checklists (L2, L3, L4 – see Table 3) and identified gaps in guiding the development and translation of narrative guidelines into computable guidelines.^6^, ^20^, ^21^ This led to developing a detailed knowledge Level 1 (L1) checklist to help guideline developers and informaticists develop clearer, more consistent, and more structured narrative guidelines for easier implementation.

**Table 3:** Knowledge levels.^3^

Knowledge level	Description	Example
L1	Narrative	Published guideline for a specific disease that is written in the format of a peer‐reviewed journal article
L2	Semi‐Structured	Flow diagram, decision tree, or other similar format that describes recommendations for implementation
L3	Structured	Standards‐compliant specification encoding logic with data model(s), terminology/code sets, value sets that is ready to be implemented
L4	Executable	CDS that is implemented and used in a local execution environment (e.g., CDS i.e. live in an EHR production system)

The L1 checklist facilitates key information (e.g., metadata, evidence, and recommendations) to successfully implement in computable formats, such as CDS systems. The hypothesis is that this solution could improve patient outcomes through evidence‐based medicine in a learning health system based on Findable, Accessible, Interoperable, and Reusable (FAIR) principles and cover clinical practice guidelines and their related systematic reviews, evidence reports, and supplementary data and materials. The draft L1 Checklist was vetted with guideline developers, informaticists, clinicians, and patients by requesting input on a published preprint and by including and citing the same preprint in the CPG‐on‐FHIR® ballot in January 2024.^22^, ^23^ Feedback was incorporated into the L1 checklist, which the team plans to publish as an extension to the GIN‐McMaster Checklist to guide development of narrative guidelines that are more readily made computable.^21^

#### 2.3.4 Marketplace team

The Marketplace Team focused on ‘*How might we* develop a solution to improve interactions and engagement of guideline development organizations (GDOs), guideline developers, and informaticists to advance understanding of the value of producing computable guidelines’. To understand challenges and opportunities in facilitating better engagement between informaticists and guideline developers, the team met with experts in both areas. They created and distributed anonymous surveys targeting GDOs and informaticists. Responses from the surveys (11 from GDOs and 11 from informaticists) plus input from other teams informed the team's proposal to establish a collaborative to connect guideline developers, implementers (those responsible for putting guidelines into practice), and informaticists (specialists in health information) called ‘Marketplace Town Square’, initially including three tracks.

First track focuses on ‘matchmaking’ GDOs with implementers and informaticists, with computable guideline test sites and testers, and with investors or funders. Establishing connections between GDOs, implementers, and informaticists is imperative to ensure guidelines are clear and can be seamlessly integrated into healthcare systems. Connecting computable guideline test sites with skilled testers is crucial for validating and refining guidelines. Testers, often informaticists with expertise in software testing, play a vital role in identifying potential issues, ensuring interoperability, and validating functionality of computable guidelines to inform clarity and utility of the narrative guidelines.

Second track focuses on access to training and technical support. Equipping GDOs with training on computable guidelines is fundamental to enhancing capacity to produce clinically sound and technologically viable guidelines. Training programs could cover principles of computable guidelines, interoperability standards, and utilization of health informatics tools, helping GDOs understand integration of technology into guideline development processes. The same applies to informaticists and implementers who are crucial in bridging the gap between clinical knowledge and technological implementation, helping them understand nuances in scientific evidence.

Third track focuses on supporting GDO leadership who navigate complexities of the evolving healthcare landscape on strategic planning that includes computable guidelines. This involves establishing a roadmap outlining key milestones, resource allocation, and clear vision for integration of computable guidelines into healthcare systems for their respective clinical or public health domains.

The team pitched AHRQ to further develop the ‘Marketplace Town Square’, emphasizing connections with AHRQ's CDS Connect, Center for Evidence and Practice Improvement (CEPI) Evidence Discovery And Retrieval (CEDAR) Project, and Systematic Review Data Repository™ (SRDR+) efforts.^24^, ^25^, ^26^, ^27^ AHRQ is considering the information provided and may request additional discussion as needed.

#### 2.3.5 AI recommendations team

The AI Recommendations Team developed a set of recommendations to encourage guideline developers to use AI to facilitate guideline development, dissemination, availability, and incorporation in clinician and patient health management workflow. There is potential to improve adherence, evaluate outcomes, and capture new evidence to enhance guideline recommendations and maintain their relevance, though the field is relatively nascent. The team created a six‐stage framework for introducing AI in the guideline continuum: evidence synthesis, authoring recommendations, personalization, clinical decision support, guideline adherence monitoring, and continuous updating of guidelines. The team plans to publish a peer‐reviewed journal article proposing AI recommendations for the guideline development community.

The outputs of the teams participating in the GIN‐NA HCD effort are summarized in Table 4.

**Table 4:** Summary of GIN‐NA HCD Team Products, Pitches, and Outcomes.

	Product(s)	Pitch(es)	Outcome
Patient‐facing/generated recommendations	Patient‐Centered Data Classes & Elements for Implementation & Decision‐Making	AHRQ	AHRQ considering info provided; additional discussions as needed
Real world testing/feedback	Proposed pilots (1) Using a platform to capture feedback through public comment during development (2) Using a platform for guideline monitoring post implementation (3) HL7® Connectathon/query building and testing	IDSA (1, 2 & 3), World Health Organization (WHO)/CDC DGHT (1 & 2), NCQA (3), multiple branches from CDC DCPC (3)	Pilots 1 & 2: IDSA, WHO, and CDC DGHT are figuring out logistics to pilot Pilot 3: Initial query testing has been completed at September 2023 HL7® Connectation; further testing in upcoming HL7® Connectathons
SOP	L1 Checklist/Peer‐reviewed journal article	CPG‐on‐FHIR® Team, WHO, GIN‐McMaster author; Submission to ‘Clinical and Public Health Guidelines’ Journal	L1 checklist and manuscript submitted for publication, included in CPG‐on‐FHIR® IG levels of knowledge checklists
Marketplace	‘Town Square’ Design	AHRQ	AHRQ considering info provided; additional discussions as needed
Al recommendations	Peer‐reviewed journal article	Submission to ‘Clinical and Public Health Guidelines’ Journal	Manuscript submitted for publication

## 3 DISCUSSION

Throughout this HCD effort, participants expressed improved understanding of other perspectives, thinking differently about their daily work, and planning to apply HCD to improve products they develop. Teams developed deliverables to be used separately or in combination by organizations (e.g., GDOs, governmental agencies, informatics and implementing organizations) to minimize or eliminate identified pain points in guideline development and implementation or included for further development within requirements in funding opportunities.

Despite challenges (e.g., competing priorities, short timeframe, vacations), most participants contributed consistently and felt their participation was valuable with sufficient time to develop solutions. Transparency kept teams connected. Improvements could include communicating more clearly before the workshop about post‐workshop activities and testing different ways to approach the post‐workshop phase. Workshop participation or previous HCD knowledge and experience may be required for team leads. More workshop time would allow more testing, developing, and connecting to ideas (e.g., more interviews, observations, co‐designing sessions) before post‐workshop refinement.

GIN regional communities, workgroups, or similar groups can model GIN‐NA's approach to connect, learn about, and develop multi‐perspective solutions. GIN‐NA Steering Group extended the value of a 1‐day in‐person HCD workshop into a multi‐week virtual effort, giving participants more hands‐on time to apply HCD. Though condensed, a 45‐min interactive workshop for the broader GIN community at the GIN 2023 Conference in Glasgow, Scotland, resulted in conference participants similarly expressing better understanding from different perspectives and value in learning about HCD.^28^

Long‐term impacts of solutions developed through this effort will take time, requiring more groups to apply the outputs, though using HCD has already reframed how participants approach their own work. Continuing to apply HCD principles in guideline development and implementation more broadly, rather than at the organizational implementation, could improve how evidence reaches practice.^10^, ^13^, ^14^

## DISCLOSURES

The findings and conclusions in this article are those of the authors and do not necessarily represent the official position of the US Centers for Disease Control and Prevention. The Guidelines International Network (GIN; www.g-i-n.net) is a Scottish Charity, recognised under Scottish Charity Number SC034047. The views expressed in this article are those of the authors and GIN is not liable for any use that may be made of the information presented.

## AUTHOR CONTRIBUTIONS

**Maria Michaels**: Conceptualization; data curation; formal analysis; funding acquisition; investigation; methodology; project administration; resources; supervision; validation; visualization; writing—original draft; writing—review and editing. **Chirine Chehab**: Data curation; formal analysis; investigation; validation; visualization; writing—review and editing. **Mindy Hangsleben**: Conceptualization; data curation; formal analysis; investigation; methodology; project administration; resources; validation; visualization; writing—review and editing.

## CONFLICT OF INTEREST STATEMENT

The authors declare no conflict of interest.

## ETHICS STATEMENT

Not applicable.

## Supporting information

Supporting Information.

## Data Availability

The data that support the findings of this study are available from the corresponding author upon reasonable request. The data that support the findings of this study are included within the paper.
