# Supplementary material for: Solving pain points in guideline development and implementation using human‐centered design
Source: Clin Public Health Guidel. 2025 Mar 4;2(2):e70014. doi: 10.1002/gin2.70014 (PMC13563372; doi:10.1002/gin2.70014)
Supplement: Supplementary file 1 [file GIN2-2-e70014-s001.pdf]

GIN-NA – Patient-Patient Advocate Conversation Guide

Introduction

Thank you for taking the time to speak with me today. Our team is working on exploring how might we help clinical staff decide the best course of action for a patient so they get or stay healthy.

After we complete our conversations with all the groups of clinical guidelines users, we will apply the insights at an upcoming GIN-NA event where guideline developers and implementers will create and test solutions to address pain points.

To ensure accuracy within our process, we would like to record this session. The recording will only be used to capture and validate the notes we are taking. Do you have any objection to us recording the interview?

Start Recording -> Confirm that recording is on and that they are ok with to record for note taking purposes.

Background/Rapport Building Questions

We're going to start out with some general questions about your role and then talk about some specific questions about your experience.

For Patients that are not as familiar with health systems, build more rapport by asking where they live, favorite place they have traveled, etc. This will help make the conversation a little more comfortable and then delve into the customer/stakeholder questions.

Patient Advocate/Rep

Can you tell me about your role/experience with the healthcare system?

What are you trying to accomplish? What tasks are involved?

What does success look like?

What outcomes do you not want to happen?

Customer/Stakeholder Main Research Questions around their role/job to be done

Walk me through your last visit to a clinic and/or stay in a hospital [as a patient / patient advocate]?

How are decisions made along the way?

How do you know your needs are being met?

How are you involved in the decision-making around your care?

How do you educate yourself on your health conditions?

What barriers do you encounter? What workarounds exist?

What do you dread doing? What do you avoid? Why?

How do you feel when you have left a good appointment? Bad appointment?

If you had a magic wand, describe to me what your experience would look like when you interact with the medical system?

Closing Questions

Thank you for spending the time to speak with us today! Before we sign off:

Is there anything that I haven't asked you that you want me to know?

Any other questions from the team?

Once we design a few solutions, would you be willing to meet again and provide feedback?
